# Prospective study of a molecular selection profile for RAS wild type colorectal cancer patients receiving irinotecan-cetuximab

**DOI:** 10.1186/s12967-015-0501-5

**Published:** 2015-05-07

**Authors:** Riccardo Giampieri, Alessandra Mandolesi, Khaled M Abouelkhair, Cristian Loretelli, Michela Del Prete, Luca Faloppi, Bianconi Maristella, Ezzeldin M Ibrahim, Marina Scarpelli, Stefano Cascinu, Mario Scartozzi

**Affiliations:** Department of Medical Oncology, University Hospital of Ancona, Polytechnic University of the Marche, Ancona, Italy; Department of Pathology, University Hospital of Ancona, Polytechnic University of the Marche, Ancona, Italy; Department of Medical Oncology, International Medical Center of Jeddah, Jeddah, KSA; Department of Medical Oncology, Medical Oncology, University Hospital of Cagliari, University of Cagliari, Cagliari, Italy

**Keywords:** Prospective selection, Anti-EGFR, Cetuximab, Colorectal cancer, RAS, BRAF, HER-3, IGF-1, PIK3CA

## Abstract

**Background:**

The aim of our study was to evaluate whether a panel of biomarkers, prospectively analysed might be able to predict patients’ clinical outcome more accurately than RAS status alone.

**Methods:**

K-RAS (exons 2, 3, 4) wild type colorectal cancer patients, candidates to second/third-line cetuximab with chemotherapy were prospectively allocated into 2 groups on the basis of their profile: favourable (BRAF and PIK3CA exon 20 wild type, EGFR GCN ≥ 2.6, HER-3 Rajkumar score ≤ 8, IGF-1 immunostaining < 2) or unfavourable (any of the previous markers altered or mutated). After the introduction of N-RAS status (exons 2, 3, 4) only RAS wild type patients were considered eligible.

Primary aim was response rate (RR). To detect a difference in terms of RR among patients with an unfavourable profile (estimated around 25%) and patients with a favourable profile (estimated around 60%), with a probability alpha of 0.05 and beta of 0.05, required sample size was 46 patients. Secondary endpoints were progression free survival (PFS) and overall survival (OS).

**Results:**

Forty-six patients were enrolled. Seventeen patients (37%) were allocated to the favourable and 29 patients (63%) to the unfavourable profile. RR in the favourable and unfavourable group was 11/17 (65%) and 2/29 (7%) (p = 0.007) respectively. The favourable group also showed an improved PFS (8 months vs. 3 months, p < 0.0001) and OS (15 months vs. 6 months, p < 0.0001).

**Conclusions:**

Our results suggest that prospective selection of optimal candidates for cetuximab treatment is feasible and may be able to improve clinical outcome.

## Introduction

The RAS (K-RAS and N-RAS) molecular testing represented a further step towards a more accurate selection of metastatic colorectal cancer patients clinically candidates to receive treatment with anti-EGFR monoclonal antibodies. Data from recent first-line trials strengthened the idea that anti-EGFR targeted agents could positively affect natural history of metastatic colorectal cancer but only when the appropriate clinical and molecular selection is applied [1-6].

As a consequence we are now able to exclude from anti-EGFR treatment more patients with potentially refractory colorectal tumours, but on the other hand we are still unable to select responding patients among those presenting with a RAS wild type status. In fact clinical observations suggested that a large proportion of patients, ranging from 40% to 60%, did not benefit from the use of anti-EGFR targeted antibodies although in the absence of a K-RAS/N-RAS mutation and are then exposed to unnecessary toxicity [7,8].

The main beyond-RAS research areas explored in the attempt to improve patients’ selection focused on the EGFR itself, the EGFR-downstream signalling pathway and the interaction between other receptors such as the IGF-1R and HER-3 [7]. Previous findings indicated that EGFR gene copy number (GCN) correlated with clinical outcome during anti-EGFR treatment in colorectal cancer patients. Many factors prevented the use of the EGFR GCN into clinical practice, particularly the inconsistency of different cut-off values from different studies [9-12].

Translational findings about growth factors receptors interdependence supported the hypothesis that HER-3 and the Insulin-like growth factor-1 (IGF-1) might affect the biological activity of the EGFR through a molecular interference with the EGFR lateral signalling ultimately determining resistance to anti-EGFR treatment [13-16]. Among other biological factors affecting the EGFR downstream pathway B-RAF mutational status and, t a lesser extent, PIK3CA mutational status resulted strongly implicated.

Many analyses indicated that B-RAF mutation might have a prognostic role although with an uncertain predictive value. Therefore the use of B-RAF for anti-EGFR treatment is indefinite and mainly based on clinicians judgement [17-21]. Notably the proportion of colorectal cancer patients potentially presenting with a B-RAF gene mutation is not negligible (about 10-15% in a K-RAS wild type population) and even proportionally increasing in an all-RAS wild type population.

Currently also mutations at exon 20 of PIK3CA, although rarely found in colorectal cancer patients (less than 5% in most studies) have been demonstrated to determine resistance to anti-EGFR monoclonal antibodies [19].

Although promising, none of these molecular markers entered clinical practice mainly because of the lack of a prospective validation.

The aim of our study was to evaluate whether a panel of molecular biomarkers including EGFR GCN, HER-3, IGF-1, B-RAF and PIK3CA prospectively analysed at the start of treatment, might be able to predict colorectal cancer patients clinical outcome during second- third-line treatment with cetuximab in combination with chemotherapy more accurately than RAS status alone.

## Patients and methods

### Patients selection and study design

All study procedures have been approved by our institutional review board (Institutional Review Board of the University Hospital of Ancona, Polytechnic University of the Marche, Ancona, Italy). Patients with histologically proven K-RAS wild type metastatic colorectal cancer, with clinical indication to receive second or third-line treatment with cetuximab in combination with chemotherapy were eligible for our study. After the introduction of N-RAS analysis only RAS wild type patients were considered for the study and all patients already included were re-analysed for N-RAS mutations. All consecutive patients with confirmed diagnosis of metastatic colorectal cancer were screened after signing informed consent to study procedure. All patients enrolled had measurable metastatic disease. Other inclusion/exclusion criteria were those commonly applied in prospective trials (i.e. adequate liver and renal function, age > 18 years).

Our primary aim was to verify whether a beyond RAS prospective molecular selection was able to improve patients outcome in terms of response rate (RR, primary endpoint of the study). Patients were prospectively allocated into 2 groups on the basis of their biomarkers profile: favourable (BRAF V600E and PIK3CA exon 20 wild type, EGFR FISH GCN ≥ 2.6, HER-3 Rajkumar score ≤ 8, IGF-1 immunostaining < 2) and unfavourable (any of the previous markers altered or mutated). To detect a difference in terms of RR among patients with an unfavourable profile (estimated around 25%) and patients with a favourable profile (estimated around 60%), with a probability alpha of 0.05 and beta of 0.05, required sample size was 46 patients. Response rate was evaluated every 8 weeks according to the Response Evaluation Criteria in Solid Tumours (RECIST, v. 1.1) by treating physicians who were blind to biomarkers results and prognostic group allocation.

### Secondary endpoints were progression free survival (PFS) and overall survival (OS)

**Figure 1:**
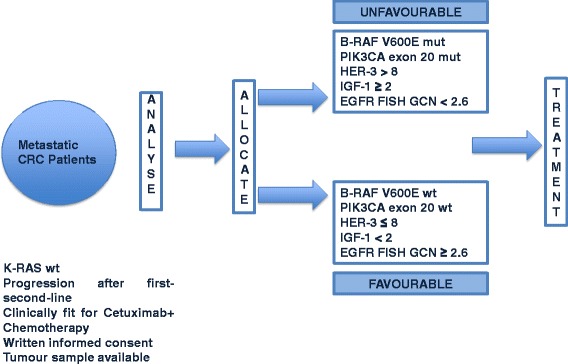
Study design. K-RAS (and then RAS) wild type colorectal cancer patients candidates to receive treatment with cetuximab were prospectively assigned to the favourable or unfavourable group on the basis of their molecular profile. All patients were then treated with cetuximab in combination with chemotherapy. Treating physicians were blind to patients allocation. RR was the primary endpoint of the study.

Overall survival and progression-free survival were defined respectively as the interval between the start of cetuximab and irinotecan therapy to death or last follow-up visit and as the interval between the start of cetuximab and irinotecan therapy to clinical progression or death or last follow up visit if not progressed.Study design is depicted in Figure 1.

Statistical analysis was performed with MedCalc software version 10.4.8 for Windows.The association between categorical variables was estimated by chi square test. Survival probability over time was estimated by the Kaplan–Meier method. Significant differences in the probability of survival between the strata were evaluated by log-rank test.

#### K-RAS mutational analysis

The variants of codons 12, 13, 59, 61, 117 and 146 of the K-RAS gene (G12D, G12A, G12V, G12S, G12R, G12C, G13D, G13C in exon 2; A59P, A59T, A59S, A59G, A59E, A59V, Q61K, Q61E, Q61R, Q61L, Q61P, Q61H in exon 3; K117Q, K117E, K117T, K117I, K117R, K117N, A146P, A146T, A146S, A146G, A146E, A146V in exon 4) have been analysed via pyrosequencing using the “Anti-EGFR MoAb response kit (KRAS status) (Diatech Pharmacogenetics., Italy).

#### N-RAS mutational analysis

The variants of codons 12, 13, 58, 59, 61, 117 and 146 of the N-RAS gene (G12D, G12A, G12V, G12S, G12R, G12C, G13R, G13S, G13A, G13V, G13D, G13C in exon 2; T58A, T58P, T58S, T58I, A59P, A59T, A59S, A59G, A59D, A59V, Q61K, Q61E, Q61R, Q61L, Q61P, Q61H in exon 3; K117Q, K117E, K117T, K117M, K117R, K117N,A146P, A146T, A146S, A146G, A146D e A146V in exon4.) have been analysed via pyrosequencing using the “Anti-EGFR MoAb response kit (N-RAS status) (Diatech Pharmacogenetics, Italy).

#### B-RAF mutational analysis

The variants of codons 464, 466, 469, and 600 (G464V, G464E, G466V, G466A, G466E, G466R, G469V, G469A, G469E, G469R in exon 11 and V471F, T599I, V600E, K601E, V600M in exon 15) have been analysed via pyrosequencing using the “Anti-EGFR MoAb response kit (BRAF status) (Diatech Pharmacogenetics, Italy). Only the V600E mutation was considered relevant to study procedure.

#### PIK3CA mutational analysis

The variants of codons 542, 545, 546 1043, 1047 and 1049 (E542K, E545K, E545A, E545G, Q546K, Q546E in exon 9 and M1043I, H1047Y, H1047R, H1047L, G1049R and G1049S in exon 20) have been analysed via pyrosequencing using the “Anti-EGFR MoAb response kit (PIK3CA status) (Diatech Pharmacogenetics, Italy). Only mutations in exon 20 were considered relevant to study procedure

#### HER-3 expression

HER-3 was evaluated with an immunohistochemical technique on 3–5 μm thick tissue sections obtained from paraffin-embedded using the monoclonal mouse antibody anti-human HER-3 (DAK-H3-IC) as previously described.

The stained slides were evaluated according to the Rajkumar score. The cut-off point with the highest sensitivity and specificity for estimating HER-3 expression was set at ≤ 8 according to previous reports [13,21].

#### IGF-1 expression

IGF-1 was evaluated with an immunohistochemical technique on 3–5 μm thick tissue sections obtained from paraffin-embedded samples using the rabbit polyclonal antibody raised against amino acids 49–118 of IGF-1 of human origin, (Santa Cruz Biotechnology) as previously reported.

IGF-1 expression was estimated by semi -quantitative analysis and classified into one of four grades. The tumour was defined positive for IGF-1 expression when the grade of the immunostaining was ≥ 2, according to previous reports [14,21].

#### Fluorescence in situ hybridization analysis for EGFR gene copy number

Fluorescence in situ hybridization (CISH) for the EGFR gene was performed using the standard dual-colour EGFR Spectrum Orange™/CEP7® Spectrum Green™ probe (Visys, Downers Grove, IL USA). The cut-off point with the highest sensitivity and specificity for estimating FISH EGFR GCN was set at 2.6 according to previous reports [9,21].

## Results

From April 2012 to May 2014, 46 patients were enrolled, 30 males (65%) and 16 females (35%), median age at study entry was 67 years. Most of the patients had an Eastern Cooperative Oncology Group Performance Status (ECOG PS) 0 or 1 (29 patients, 63%) and 2 or more metastatic sites (39 patients, 85%). Thirty-six patients (78%) received at least 2 previous lines of treatment. In the whole population we observed 13 partial remissions (26%), median PFS was 3.3 months and median OS was 11 months. Main patients’ characteristics have been summarised in Table 1. Overall we found 2 BRAF V600E mutations (4%) and 3 PIK3CA exon 20 mutations (6%). The remaining patients were wild type for either B-RAF or PIK3CA mutational status. EGFR FISH GCN was ≥ 2.6 in 28 patients (61%), HER-3 Rajkumar score was ≤ 8 in 25 patients (56%), IGF-1 immunostaining was < 2 in 30 patients (65%) (Table 2).

**Table 1:** **Patients characteristics and main study results according to the 2 study groups (unfavourable and favourable)**

	**Whole group**	**Unfavourable**	**Favourable**	***p*** **value**
	**(n = 46)**	**(n = 29)**	**(n = 17)**	
**Age (range)**	67 (36–81)	66 (36–79)	67 (38–81)	ns
**Sex**				
Males	30 (65%)	21 (72%)	9 (53%)	ns
Females	16 (35%)	8 (28%)	7 (47%)	ns
**ECOG PS**				
0-1	29 (63%)	18 (62%)	11 (65%)	ns
2-3	17 (47%)	11 (38%)	6 (35%)	ns
**Metastatic sites**				
1	7 (15%)	5 (17%)	2 (12%)	ns
≥ 2	39 (85%)	24 (83%)	15 (88%)	ns
**Previous lines of treatment**				
1	10 (22%)	6 (21%)	4 (9%)	ns
≥ 2	36 (78%)	23 (89%)	15 (88%)	ns
**Treatment**				
FOLFIRI/Cet	9 (19%)	6 (21%)	3 (23%)	ns
Irinotecan/Cet	37 (81%)	23 (79%)	14 (77%)	ns
**Response rate**				
PR	13 (26%)	2 (7%)	11 (65%)	0.007
SD	6 (16%)	3 (10%)	3 (18%)	0.8
DCR (PR + SD)	19 (41%)	5 (17%)	14 (82%)	0.0001
PD	27 (59%)	24 (83%)	3 (18%)	0.0001
**Survival**				
PFS(months)	3.3	3	8	< 0.0001
OS (months)	11	6	15	< 0.0001

ECOG PS = Eastern Cooperative Oncology Group Performance Status.

FOLFIRI = Fluorouracil, leucovorin, Irinotecan.

Cet = Cetuximab.

PR = Partial Response.

SD = Stable Disease.

DCR = Disease Control Rate.

PD = Progressive Disease.

**Table 2:** **Results for molecular markers analysed**

	**Whole group (n = 46)**
**BRAFV600E**	
Mut	2 (4%)
Wild Type	44 (96%)
**PIK3CA exon 20**	
Mut	3 (6%)
Wild Type	43 (94%)
**EGFR FISH GCN**	
<2.6	18 (39%)
≥2.6	28 (61%)
**HER 3 IHC Score**	
>8	20 (44%)
≤8	26 (56%)
**IGF-1 IHC Score**	
≥2	16 (35%)
<2	30 (65%)

N-RAS analysis of patients enrolled before the introduction of N-RAS wild type status as a further prerequisite for anti-EGFR treatment (35 patients) revealed 1 N-RAS mutation (3%) in a patient allocated to the unfavourable group because of HER-3 over-expression.

According to biomarkers results 17 patients (37%) were prospectively allocated to the favourable group and 29 patients (63%) were prospectively allocated to the unfavourable group. Main clinical characteristics (age, sex, ECOG PS, previous treatments, number of metastatic sites) resulted comparable between the 2 groups (favourable vs. unfavourable) (Table 1). Patients with the unfavourable profile showed 2 BRAF mutations, 3 PIK3CA exon 20 mutations, 18 cases of FISH EGFR GCN < 2.6, 20 cases of HER-3 and 16 cases of IGF-1 overexpression respectively.

In the favourable group we observed 11 patients (65%) with a partial response (PR), whereas in the unfavourable group only 2 patients (7%) showed a PR (p = 0.007).

Disease control rate (DCR) resulted also improved in the favourable group (82% vs. 17%, p = 0.0001). Accordingly only 3 patients (18%) in the favourable group and as much as 24 patients (83%) in the unfavourable group progressed during treatment (p = 0.0001) (Table 1).

**Figure 2:**
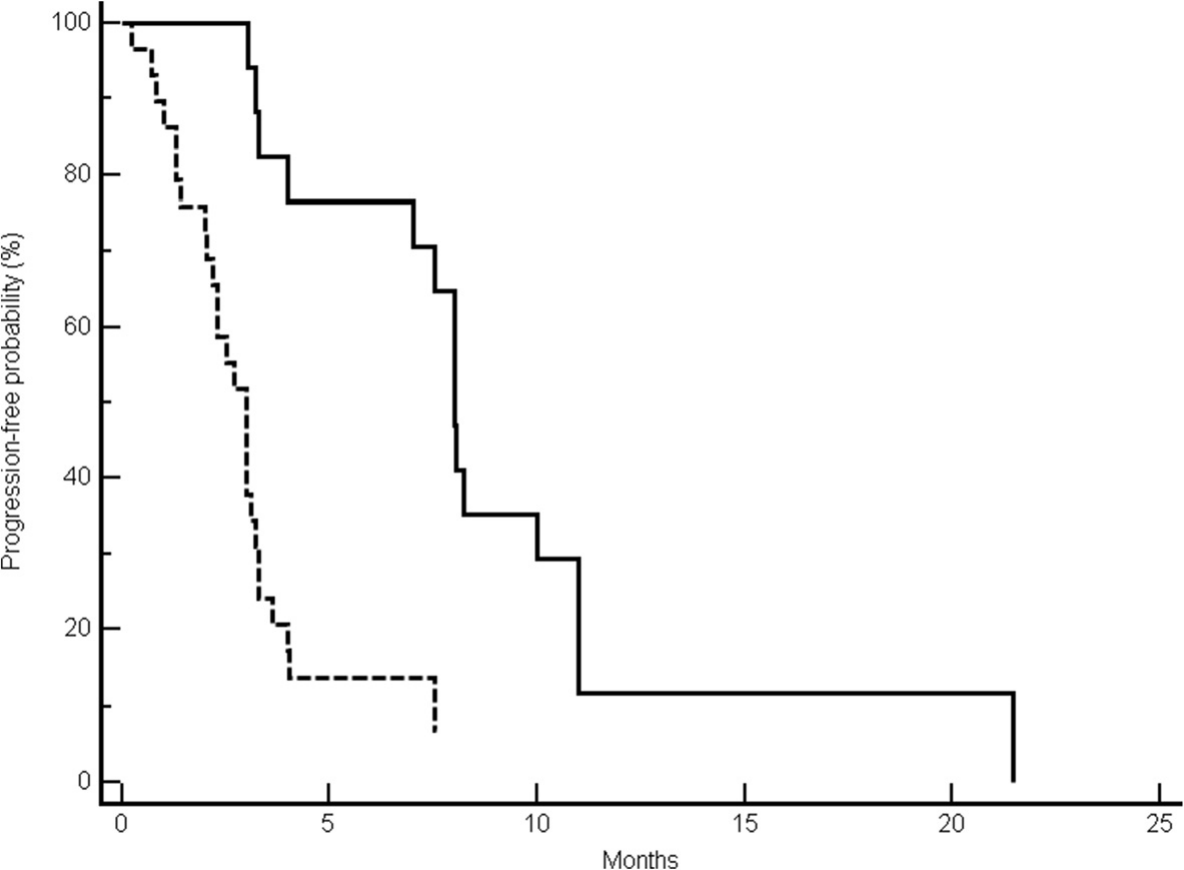
Median progression-free survival (PFS) results. Kaplan-Meier curves for median PFS of colorectal cancer patients treated with irinotecan and cetuximab prospectively allocated in the unfavourable (-------) and favourable (———) arm (3 months vs. 8 months, *p* < 0.0001).

**Figure 3:**
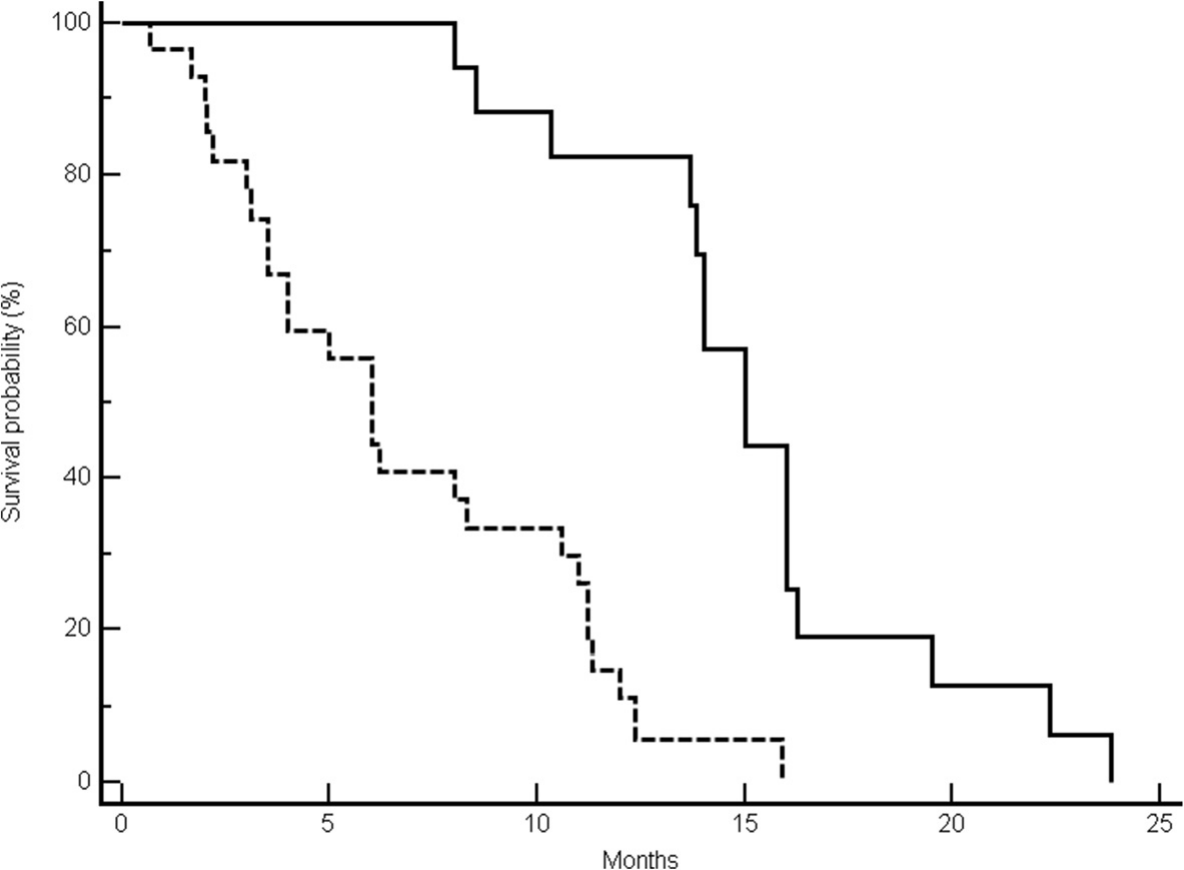
Median overall survival (OS) results. Kaplan-Meier curves for median OS of colorectal cancer patients treated with irinotecan and cetuximab prospectively allocated in the unfavourable (-------) and favourable (———) arm (6 months vs. 15 months, *p* < 0.0001).

Median PFS was 8 months in the favourable group, whereas it was 3 months in the favourable group (p < 0.0001) (Figure 2). Median OS in the favourable and unfavourable group was 15 months vs. 6 months respectively (p < 0.0001) (Figure 3).

## Discussion

Many years after the introduction of anti-EGFR monoclonal antibodies the debate about the optimal molecular and clinical selection is more current than ever. Although the introduction of RAS mutational analysis improved our ability to exclude resistant patients, there is still a clear clinical need of further refining the selection process.

At present the beyond RAS molecular selection for monoclonal antibodies directed against the EGFR is one of the most actively investigated topic. However the final impact on clinical practice of research findings appears clearly not proportionate to the vast amount of data produced.

We suggest that a beyond-RAS prospective selection is feasible and potentially effective in further identifying optimal candidates for anti-EGFR treatment. We believe that the observed 65% response rate observed in the favourable group might be of great interest particularly in a pre-treated population where RR with anti-EGFR monoclonal antibodies is usually reported at around 20-40% (depending on the treatment line). Accordingly the disappointing RR found in the unfavourable group (7%) is a suggestion of an inadequate clinical activity in this group of RAS wild type patients.

The response rate in this prospective, biologically enriched study is in line with the study hypothesis and comparable with results from retrospective studies investigating these biological markers [22].

In retrospective series response rate for K-RAS wild type patients showing HER-3 and IGF-1 negative tumours ranged in fact from 50 to 65%. Similarly a response rate ranging from 30 to 88% was observed among patients with increased EGFR GCN.

On the contrary several analyses were concordant in fixing at around 0% the RR for B-RAF mutant tumours [18-20]. Very similar figures have been reported for PIK3CA mutant tumours [19].

Although survival parameters were not primary aims of our study, we also suggested that a more accurate molecular selection might have a significant impact also on PFS and OS.

The effect on PFS and OS was however less pronounced than that reported in previous retrospective analyses. The prospective selection itself might have reduced the magnitude of the overall survival results observed in retrospective studies. More importantly the study was not designed to detect a difference in median PFS or OS and therefore survival results are merely speculative.

## Conclusions

The present study represents the proof of principle that a prospective molecular selection is feasible and clinically relevant. On the one hand we demonstrated that a beyond-RAS molecular profile might improve the therapeutic efficacy of cetuximab in the favourable group of patients, while on the other hand we also suggested that in the unfavourable group of patients anti-EGFR strategies are ineffective. This implies that different treatment options should be pursued in these patients.

A straightforward interpretation of our findings should be regarded as scientifically difficult. At this point it is not in fact possible to define the individual and independent role of each biological variable analysed. The patients’ number was clearly not planned for this aim and it is inadequate for further speculations.

At our knowledge this is the first prospectively designed study with the specific aim to define the role of a molecular profile in metastatic colorectal cancer patients receiving cetuximab.

As our patients’ expectations are understandingly increasing, any effort should be made in order to move from retrospective to prospective and ultimately into practice in order to maximise efficacy and avoid unnecessary toxicities.

## References

[1] Van Cutsem E, Kohne CH, Hitre E, Zaluski J, Chang Chien CR, Makhson A (2009). Cetuximab and chemotherapy as initial treatment for metastatic colorectal cancer. N Engl J Med.

[2] Douillard JY, Oliner KS, Siena S, Tabernero J, Burkes R, Barugel M (2008). Panitumumab-FOLFOX4 treatment and RAS mutations in colorectal cancer. N Engl J Med.

[3] Bokemeyer C, Van Cutsem E, Rougier P, Ciardiello F, Heeger S, Schlichting M (2012). Addition of cetuximab to chemotherapy as first-line treatment for KRAS wild-type metastatic colorectal cancer: pooled analysis of the CRYSTAL and OPUS randomised clinical trials. Eur J Cancer.

[4] Cunningham D, Humblet Y, Siena S, Khayat D, Bleiberg H, Santoro A (2004). Cetuximab monotherapy and cetuximab plus irinotecan in irinotecan-refractory metastatic colorectal cancer. N Engl J Med.

[5] Jonker DJ, O’Callaghan CJ, Karapetis CS, Zalcberg JR, Tu D, Au HJ (2007). Cetuximab for the treatment of colorectal cancer. N Engl J Med.

[6] Amado RG, Wolf M, Peeters M, Van Cutsem E, Siena S, Freeman DJ (2008). Wild-Type KRAS Is Required for Panitumumab Efficacy in Patients With Metastatic Colorectal Cancer. J Clin Oncol.

[7] Giampieri R, Scartozzi M, Del Prete M, Maccaroni E, Bittoni A, Faloppi L (2013). Molecular biomarkers of resistance to anti-EGFR treatment in metastatic colorectal cancer, from classical to innovation. Crit Rev Oncol Hematol.

[8] Giampieri R, Aprile G, Del Prete M, Faloppi L, Bianconi M, Bonotto M (2014). Beyond RAS: the role of epidermal growth factor receptor (EGFR) and its network in the prediction of clinical outcome duringanti-EGFR treatment in colorectal cancer patients. Curr Drug Targets.

[9] Scartozzi M, Bearzi I, Mandolesi A, Pierantoni C, Loupakis F, Zaniboni A (2009). Epidermal Growth Factor Receptor (EGFR) gene copy number (GCN) correlates with clinical activity of irinotecan-cetuximab in K-RAS wild-type colorectal cancer: a fluorescence in situ (FISH) and chromogenic in situ hybridization (CISH) analysis. BMC Cancer.

[10] Moroni M, Veronese S, Benvenuti S, Marrapese G, Sartore-Bianchi A, Di Nicolantonio F (2005). Gene copy number for epidermal growth factor receptor (EGFR) and clinical response to anti-EGFR treatment in colorectal cancer: a cohort study. Lancet Oncol.

[11] Sartore-Bianchi A, Moroni M, Veronese S, Carnaghi C, Bajetta E, Luppi G (2007). Epidermal growth factor receptor gene copy number and clinical outcome of metastatic colorectal cancer treated with panitumumab. J Clin Oncol.

[12] Cappuzzo F, Finocchiaro G, Rossi E, Jänne PA, Carnaghi C, Calandri C (2008). EGFR FISH assay predicts for response to cetuximab in chemotherapy refractory colorectal cancer patients. Ann Oncol.

[13] Scartozzi M, Mandolesi A, Giampieri R, Bittoni A, Pierantoni C, Zaniboni A (2011). The role of HER-3 expression in the prediction of clinical outcome for advanced colorectal cancer patients receiving irinotecan and cetuximab. Oncologist.

[14] Scartozzi M, Mandolesi A, Giampieri R, Pierantoni C, Loupakis F, Zaniboni A (2010). Insulin-like growth factor 1 expression correlates with clinical outcome in K-RAS wild type colorectal cancer patients treated with cetuximab and irinotecan. Int J Cancer.

[15] Finocchiaro G, Cappuzzo F, Rossi E, Toschi M, Janne PA, Roncalli M, et al. Insulin-like growth factor receptor-1 (IGFIR), MET and BRAF and primary resistance to cetuximab therapy in colorectal cancer patients. J Clin Oncol. 2008; 26 (suppl). abstract 4135.

[16] Winder T, Zhang W, Yang D, Ning Y, Bohanes P, Gerger A (2010). Germline polymorphisms in genes involved in the IGF1 pathway predict efficacy of cetuximab in wild-type KRAS mCRC patients. Clin Cancer Res.

[17] Park JH, Han SW, Oh DY, Im SA, Jeong SY, Park KJ (2011). Analysis of KRAS, BRAF, PTEN, IGF1R, EGFR intron 1 CA status in both primary tumors and paired metastases in determining benefit from cetuximab therapy in colon cancer. Cancer Chemother Pharmacol.

[18] Di Nicolantonio F, Martini M, Molinari F, Sartore-Bianchi A, Arena S, Saletti P (2008). Wild-type BRAF is required for response to panitumumab or cetuximab in metastatic colorectal cancer. J Clin Oncol.

[19] De Roock W, Claes B, Bernasconi D, De Schutter J, Biesmans B, Fountzilas G (2010). Effects of KRAS, BRAF, NRAS, and PIK3CA mutations on the efficacy of cetuximab plus chemotherapy in chemotherapy-refractory metastatic colorectal cancer: a retrospective consortium analysis. Lancet Oncol.

[20] Laurent-Puig P, Cayre A, Manceau G, Buc E, Bachet JB, Lecomte T (2009). Analysis of PTEN, BRAF, and EGFR status in determining benefit from cetuximab therapy in Wild-Type KRAS metastatic colon cancer. J Clin Oncol.

[21] Van Cutsem E, Köhne CH, Láng I, Folprecht G, Nowacki MP, Cascinu S (2011). Cetuximab plus irinotecan, fluorouracil, and leucovorin as first-line treatment for metastatic colorectal cancer: updated analysis of overall survival according to tumor KRAS and BRAF mutation status. J Clin Oncol.

[22] Scartozzi M, Giampieri R, Maccaroni E, Mandolesi A, Giustini L, Silva RR (2012). Analysis of HER-3, insulin growth factor-1, nuclear factor-kB and epidermal growth factor receptor gene copy number in the prediction of clinical outcome for K-RAS wild-type colorectal cancer patients receiving irinotecan-cetuximab. Ann Oncol.

